# Improving Healthcare Professionals’ Access to Addiction Medicine Education Through VHA Addiction Scholars Program

**DOI:** 10.5811/westjem.17850

**Published:** 2024-05-20

**Authors:** Zahir Basrai, Manuel Celedon, Nathalie Dieujuste, Julianne Himstreet, Jonathan Hoffman, Cassidy Pfaff, Jonie Hsiao, Robert Malstrom, Jason Smith, Michael Radeos, Terri Jorgenson, Melissa Christopher, Comilla Sasson

**Affiliations:** * VA Greater Los Angeles Health Care System, Veterans Health Administration, Department of Emergency Medicine, Los Angeles, California; † VA Eastern Colorado Health Care System, Veterans Health Administration, Aurora, Colorado; ‡ University of Colorado, Anschutz Medical Campus, Aurora, Colorado; § VA Pharmacy Benefits Management Academic Detailing Service, Eugene, Oregon; ∥ VA VISN 19 Rocky Mountain Network, Salt Lake City, Utah; ¶ VISN 19 Academic Detailing Service, Veterans Health Administration, Tulsa, Oklahoma; # VA Pharmacy Benefits Management Academic Detailing Service, Martinez, California; ** VISN 19 Academic Detailing Service, Veterans Health Administration, Denver, Colorado; †† NYC Health + Hospitals/Coney Island, Department of Emergency Medicine, Brooklyn, New York; ‡‡ Pharmacy Benefits Management, Clinical Pharmacy Practice Office, Washington, DC; §§ VA Pharmacy Benefits Management Academic Detailing Service, San Diego, California

## Abstract

**Introduction:**

The seemingly inexorable rise of opioid-related overdose deaths despite the reduced number of COVID-19 pandemic deaths demands novel responses and partnerships in our public health system’s response. Addiction medicine is practiced in a broad range of siloed clinical environments that need to be included in addiction medicine training beyond the traditional fellowship programs. Our objective in this project was to implement a knowledge-based, live virtual training program that would provide clinicians and other healthcare professionals with an overview of addiction, substance use disorders (SUD), and clinical diagnosis and management of opioid use disorder (OUD).

**Methods:**

The Veterans Health Administration (VHA) Emergency Department Opioid Safety Initiative (ED OSI) offered a four-day course for healthcare professionals interested in gaining knowledge and practical skills to improve VHA-based SUD care. The course topics centered around the diagnosis and treatment of SUD, with a focus on OUD. Additionally, trainees received six months of support to develop addiction medicine treatment programs. Evaluations of the course were performed immediately after completion of the program and again at the six-month mark to assess its effectiveness.

**Results:**

A total of 56 clinicians and other healthcare professionals participated in the Addiction Scholars Program (ASP). The participants represented nine Veteran Integrated Service Networks and 21 different VHA medical facilities. Nearly 70% of participants completed the initial post-survey. Thirty-eight respondents (97.4%) felt the ASP series contained practical examples and useful information that could be applied in their work. Thirty-eight respondents (97.4%) felt the workshop series provided new information or insights into the diagnosis and treatment of SUD. Eleven capstone projects based on the information acquired during the ASP were funded (a total of $407,178). Twenty participants (35.7%) completed the six-month follow-up survey. Notably, 90% of respondents reported increased naloxone prescribing and 50% reported increased prescribing of buprenorphine to treat patients with OUD since completing the course.

**Conclusion:**

The ASP provided healthcare professionals with insight into managing SUD and equipped them with practical clinical skills. The students translated the information from the course to develop medication for opioid use disorder (M-OUD) programs at their home institutions.

## INTRODUCTION

The national opioid epidemic is one of the leading preventable causes of morbidity and premature death in the United States. In 2017, the US Department of Health and Human Services (HHS) declared the opioid crisis a public health emergency.^1^ The COVID-19 pandemic has exacerbated this crisis with an increased prevalence of opioid use disorder (OUD) and deaths from prescription and non-prescription opioids.^2^ Veterans are at nearly twice the risk of fatal drug overdose when compared to non-veterans.^3^ As part of the five priorities to combat the opioid crisis HHS highlighted the importance of improving access to prevention, treatment, and recovery support services.^1^ However, there remain critical shortages of healthcare professionals who can provide these life-saving services.^4^ Improving access to substance use disorder (SUD) care at any time, any place is an important part of the Veterans Health Administration’s (VHA) strategy. As a result, there is a growing need for training healthcare professionals outside the traditional addiction medicine specialty on key components of addiction medicine and SUD.

The VHA is America’s largest integrated healthcare system, providing care at 1,298 healthcare facilities including 171 medical centers and 1,113 VHA outpatient clinics. More than nine million enrolled veterans are served by the VHA each year.^5^ Despite its size, the VHA system has a shortage of addiction specialists and SUD clinics. As a result, the responsibility of providing SUD care falls on a variety of specialties, including pharmacy and mental health, and primary care and emergency medicine. However, the education opportunities for these practitioners to obtain advanced training in addiction medicine is limited.

Currently, addiction medicine is not a required graduate medical education course for internal medicine, family medicine, or emergency medicine residencies. As a result, trainees receive variable exposure to SUD care during residency, leading to suboptimal preparation managing patients with addiction when practicing independently.^6^^,^^7^ The traditional pathway for addiction medicine training is to complete a 12-month dedicated fellowship at one of the 90 sites accredited by the Accreditation Council for Graduate Medical Education.^8^ This significant commitment limits the ability for frontline clinicians to obtain further training in addiction medicine. There is a need to create accessible didactic and practical clinical education in addiction medicine to increase frontline clinician comfort.

Lack of basic training in SUD is a significant barrier to physician engagement of medication for opioid use disorder (M-OUD) programs.^9^^,^^10^ As a result, the Addiction Scholars Program (ASP) was developed to provide additional training for physician assistants, nurse practitioners, clinical pharmacists, academic detailing pharmacists, and physicians. The educational topics included a foundational understanding of the treatment of OUD, complex pain, and complex persistent opioid dependence. Our objective in this study was to measure the effectiveness, immediately and at six months, of a hybrid educational intervention paired with creation of multidisciplinary teams on knowledge retention and willingness to prescribe M-OUD.

## METHODS

This was a post-implementation study of the ASP, a novel hybrid educational approach and facilitated, team-based quality improvement (QI) project. Surveys were performed at the conclusion of the course and at the six-month mark. The surveys focused on the course’s effectiveness and the trainee’s willingness to initiate an addiction medicine project at their site. We used descriptive statistics to interpret the results of the survey. The Emergency Department Opioid Safety Initiative (ED OSI) program was designated as a QI project through the Office of Pharmacy Benefits Management Academic Detailing Service from the institutional review board of the Edward Hines, Jr. VA Hospital and approved by the Rocky Mountain Regional VA Medical Center Research and Development service.

### Addiction Scholars Program

The ASP is a part of the VHA ED OSI and was developed as an intensive course for clinicians interested in understanding VHA-based SUD care. Frontline clinicians and other healthcare professionals who were current employees of the VHA were invited to apply to attend the ASP. Forty were accepted to attend the program. The course consisted of four virtual sessions that were each four hours long. Each session covered fundamental and advanced topics of addiction medicine for emergency and acute care settings.

The entire course was delivered virtually using the Microsoft Teams (Microsoft Corp, Redmond, WA) application. Topics included clinical management of OUD, opioid overdose management, buprenorphine induction, naloxone distribution, pain management in patients with OUD, and opioid-induced chronic pain syndrome. The program used a combination of lectures and case-based breakout sessions to reinforce key concepts. Lecturers were selected based on their experience and expertise in specific areas of addiction medicine. Interdisciplinary groups were strategically assembled for the case-based breakout sessions with members from the same VHA site and Veteran Integrated Service Networks (VISN). This allowed for a networking opportunity where group members could build connections that would lead to the development of M-OUD programs locally at their VHA site or at their VISN. The groups were paired with a member of the VHA ED OSI team who would facilitate discussion of the cases.

After successful completion of the course, trainees received six months of support to develop and implement addiction medicine treatment programs. Trainees were also encouraged to submit capstone projects, which were eligible for funding up to $50,000 (up to two years) to help implement addiction medicine projects at their local VHA site.

## RESULTS

A total of 56 individuals participated in the ASP, including 32 clinicians, 10 clinical pharmacy practitioners, and 14 academic detailing pharmacists. The clinicians represented nine VISNs and 21 different VHA facilities. The class was composed of 15 physicians, seven nurses and nurse practitioners, 31 pharmacists, and three physician assistants. Participants ranged in age from 30–65 (mean 46.2 years) and had been in clinical practice for an average of 11 years (Table 1). Additionally, attendees represented numerous clinical service areas including emergency medicine, urgent care, primary care, pain management, mental health, and substance use treatment.

**Table 1.** Scholar characteristics.

	Scholars (%) (*N* = 32)
Profession	
Physician	15 (46.9)
Nurse practitioner	6 (18.8)
Nurse	1 (3.1)
Physician assistant	3 (9.4)
Pharmacist	7 (21.9)
Years out of training	
0–5 years	13 (40.6)
6–10 years	6 (18.8)
10+ years	10 (31.3)
Missing	3 (9.4)
Clinical Area	
Emergency department or urgent care	6 (18.8)
Mental health, substance use treatment, or psychiatry	14 (43.8)
Pain management	3 (9.4)
Primary care	5 (15.6)
Pharmacy	1 (3.1)
Missing	3 (9.4)

Of the 56 participants, 39 (almost 70%) responded to the initial post-survey. Thirty-eight respondents (97.4%) reported that the ASP series contained practical examples and useful information that could be applied in their work. Thirty-eight respondents (97.4%) felt that the workshop series provided new information or insights into the diagnosis and treatment of SUD. Thirty-five respondents (89.7%) were very or somewhat satisfied with the ASP series.

Twenty individuals who participated in the ASP responded to the six-month follow-up survey. The majority of respondents (85.0%) reported feeling “comfortable” or “very comfortable” initiating M-OUD since completing the ASP. Fourteen (70% of follow up respondents) pursued additional M-OUD training since completing the ASP. Of the 20 respondents, four worked in departments without an active M-OUD program; three of the four (75%) are currently working to develop an M-OUD program. Eighteen (90%) of the respondents reported increased naloxone prescribing since completing the ASP. Ten (50%) of the respondents increased prescribing of buprenorphine to treat patients with OUD since completing the course (Table 2).

**Table 2.** Results of initial and six-month follow-up survey.

	Initial follow-up (N = 39)
The ASP series contained practical examples and useful information that can be applied in their work.	38 (97.4%)
The workshop series provided new information or insights into the diagnosis and treatment of SUD.	38 (97.4%)
“Very” or “somewhat” satisfied with the ASP series.	35 (89.7%)
	6-month follow-up (n = 20)
“Comfortable” or “very comfortable” initiating M-OUD since completing the ASP.	17 (85%)
Pursued additional M-OUD training since completing the ASP.	14 (70%)
Work in departments without an active M-OUD program.	4 (20%)
Increased naloxone prescribing since completing the ASP.	18 (90%)
Increased prescribing of buprenorphine to treat patients with OUD since completing the ASP.	10 (50%)

*ASP*, Addiction Scholars Program; *SUD*, substance use disorder; *OUD*, opioid use disorder; *M-OUD*, medication for opioid use disorder.

At the conclusion of the ASP, 11 capstone projects were submitted and awarded a total of $407,178. Seven (63.6%) of the projects focused on the development of naloxone or buprenorphine programs. Other projects were focused on harm reduction with the development of a syringe service program, the use of fentanyl testing strips, development of a VISN-wide virtual learning program for SUD training, urine point-of-care testing for controlled medications, and music- and movement-based interventions to engage high-risk veterans in substance use treatment.

## DISCUSSION

Our study demonstrated the ASP successfully provided additional addiction medicine training to clinicians and other healthcare professionals and that there is a desire for additional addiction medicine training within the VHA system. The ASP was designed as an educational program with an emphasis on promoting facility-level team building to enhance cross-functional clinical care. These findings are encouraging as, after completing the ASP, healthcare professionals without formal addiction medicine training were able to advocate for OUD treatment in non-SUD specialty clinical settings at their local VHA site. Successful treatment of patients with OUD requires a multidisciplinary approach involving both the addiction medicine service and the outpatient primary care team. Empowering non-SUD specialty clinics with the knowledge and practical skills to treat OUD is essential in implementing the “no wrong door” approach to OUD treatment.^11^ The support and networking opportunities provided by the ASP successfully led to the development of local addiction medicine programs at VHA sites as evidenced by the 11 capstone projects that were funded.

The success of the ASP was due in part to the blended learning structure of the course. Lectures were curated and delivered by experts in the field and ranged from basic addiction medicine topics to more advanced topics. This allowed for engagement of all learners regardless of their specialty or level of training. The course also leveraged a team-based learning approach through the breakout sessions, which reinforced key components of treating complex patients with OUD. Team-based learning has been shown to have positive outcomes for students in terms of student experience.^12^

The e-learning platform also allowed for engagement by a wider audience than would have otherwise been possible by an in-person course. The ASP gave additional addiction medicine training to those who would otherwise not have been eligible for a fellowship by the traditional pathway. This allowed for engagement of key stakeholders who could implement programs at local facilities in areas that are separate from dedicated SUD clinics. The ASP is a scalable program that can be further developed and replicated outside of the VHA system.

## LIMITATIONS

Although the program did receive favorable ratings, it is important to note that attendees did self-select to attend; as a result, they may have been more biased in their ratings of an addiction medicine program. Future efforts will be made to recruit clinicians and other healthcare professionals who may be resistant or hesitant to the addition of substance use and opioid safety measures in their practice. Further studies are needed to assess actual interest in additional addiction medicine training throughout the VHA system. It should be noted, too, that this study provided only a six-month follow-up, at which point the participants’ survey response rate was low. Additionally, the results of this study are survey based, and thus the limitations that apply to surveys also apply here.

The survey did not contain knowledge-based questions to assess retention of knowledge. Future iterations of the course will contain knowledge-based questions to assess for acquisition of knowledge. Future studies will also need to look at how the ASP influenced the development of addiction medicine programs in the VHA system. Studies will also need to examine how successful the management of OUD is in nontraditional settings that are outside the SUD clinics. Future studies can also be conducted to compare long-term outcomes for patients whose healthcare professionals participated in ASP compared to those who did not.

## CONCLUSION

This feasibility study has shown that ASP equipped clinicians and other healthcare professionals with an intensive overview of addiction medicine. The students translated the information from the course to develop M-OUD programs at their home institutions.
